# Supplementary material for: MMDRP: drug response prediction and biomarker discovery using multi-modal deep learning
Source: Bioinform Adv. 2024 Jan 20;4(1):vbae010. doi: 10.1093/bioadv/vbae010 (PMC10872075; doi:10.1093/bioadv/vbae010)
Supplement: vbae010_Supplementary_Data [file vbae010_supplementary_data.zip › MMDRP_Supplementary.pdf]

Supplementary Tables and Figures

Supplementary Table 1. Targeted drugs in CTRPv2 by cancer type.

Cancer Type	Targeted Drugs
**Leukemia**	Idelalisib, Venetoclax, Tretinoin, Dasatinib, Ibrutinib, Nilotinib, Bosutinib
**Breast Cancer**	Olaparib, Tamoxifen, Lapatinib, Fulvestrant, Alpelisib
**Lung Cancer**	Crizotinib, Erlotinib, Gefitinib, Dabrafenib
**Colorectal Cancer**	Regorafenib
**Lymphoma**	Bortezomib, Bexarotene, Belinostat, Vorinostat
**Thyroid Cancer**	Cabozantinib, Vandetanib
**Skin Cancer**	Sonidegib, Trametinib, Vemurafenib
**Kidney Cancer**	Axitinib, Temsirolimus, Sorafenib
**Soft Tissue Sarcoma**	Pazopanib
**Pancreatic Cancer**	Sunitinib

Supplementary Table 2. Databases relevant to the drug response prediction task used in this project.

Resource	Data	Description
DepMap (21Q2)	**Mutation:** 18,802 genes, 1,734 cell lines (WGS)<br>**Copy Number:** 27,561 gene overlaps, 1,742 cell lines (Mostly WGS)	1,814 Cell Lines Overall (Incomplete Data)

	**Gene Expression:** 19,176 genes, 1,378 cell lines (RNA-Seq)<br>**Protein Quantification: (**up to) 12,399 proteins, 5,153 shared across all cell lines, 378 cell lines<br>**Histone Profiling:** 42 histone modification sites, 896 cell lines<br>**miRNA Expression:** 734 miRNAs, 953 cell lines<br>**Metabolomics:** 225 metabolites, 927 cell lines<br>**RPPA:** 214 proteins, 898 cell lines	
CTRPv2	**Compound**: Structure, Synonyms, Target, Concentrations<br>**Dose-Response:** 310,792 Dose-Response Values	860 cell lines (rows) by 481 drugs (columns) (incomplete)
ChEMBL[58,59] (version 27)	**Compound**: SMILES representation of drug-like molecules	1,941,411 Compounds

Supplementary Table 3. Best bimodal models and their RMSE losses by omic type, splitting method, and drug type in the AAC ≥ 0.7 range.

Omic Type	Split By Both Cell Line & Drug Scaffold		Split By Cell Line		Split By Drug Scaffold	
	Targeted Drug	Untargeted Drug	Targeted Drug	Untargeted Drug	Targeted Drug	Untargeted Drug
MUT	LDS+LMF 0.599	LMF 0.28	LDS+LMF 0.566	LMF 0.334	LDS+LMF+GNN 0.547	LDS+LMF+GNN 0.221

Omic Type	Split By Both Cell Line & Drug Scaffold		Split By Cell Line		Split By Drug Scaffold	
	Targeted Drug	Untargeted Drug	Targeted Drug	Untargeted Drug	Targeted Drug	Untargeted Drug
CNV	LMF+GNN 0.448	LMF 0.209	LMF 0.431	LDS+LMF+GNN 0.21	LDS+LMF+GNN 0.435	LDS+LMF+GNN 0.195
EXP	LMF 0.456	LMF 0.19	LMF 0.451	LMF 0.185	LDS+LMF+GNN 0.403	LDS+LMF+GNN 0.166
PROT	LMF+GNN 0.48	LDS+LMF+GNN 0.211	LMF+GNN 0.454	GNN 0.208	LDS+LMF+GNN 0.246	LDS+LMF+GNN 0.13
MIRNA	LMF+GNN 0.542	LMF 0.228	LMF 0.435	LMF 0.213	LMF 0.49	LDS+LMF+GNN 0.21
METAB	LMF+GNN 0.503	LDS+LMF 0.219	LDS+LMF+GNN 0.576	LMF 0.232	LDS+LMF+GNN 0.542	LDS+LMF+GNN 0.209
HIST	LDS+LMF+GNN 0.579	LDS+LMF+GNN 0.245	GNN 0.572	GNN 0.259	Sum 0.569	LDS+LMF+GNN 0.235
RPPA	GNN 0.524	LMF 0.204	Sum 0.484	LMF 0.209	LDS+LMF+GNN 0.489	LDS+LMF+GNN 0.214

Supplementary Table 4. Summary of top repurposable drug candidates (Empirical AAC ≥ Highest Prescribed AAC + 0.2) paired with the model that can predict the empirical AAC most accurately (MAE ≤ 0.2). Note that the empirical cell line is novel to the model.

Empirical				Prescribed			Top Model: Data Type(s) & Prediction
Primary Disease	Lineage Subtype	Cell Line	AAC	Highest AAC	Cancer	Drug	
Brain Cancer	Glioma	AM38	0.684	0.367	Breast Cancer	Alpelisib	HIST + RPPA 0.73

Empirical				Prescribed			Top Model: Data Type(s) & Prediction
**Primary Disease**	**Lineage Subtype**	**Cell Line**	**AAC**	**Highest AAC**	**Cancer**	**Drug**	
Breast Cancer	Breast Carcinoma	DU4475	0.953	0.293	Lung Cancer	Dabrafenib	MUT + EXP 0.777
		HCC1569	0.567	0.299	Kidney Cancer	Temsirolimus	METAB + RPPA 0.598
	Breast Ductal Carcinoma	HCC1954	0.631				MIRNA + HIST 0.612
Colon/Colorectal Cancer	Colorectal Adenocarcinoma	NCIH716	0.481	0.253	Sarcoma	Pazopanib	MUT + PROT 0.38
Endometrial/Uterine Cancer	Endometrial Adenocarcinoma	JHUEM2	0.564	0.299	Kidney Cancer	Temsirolimus	MIRNA + HIST 0.559
Head and Neck Cancer	Upper Aerodigestive Squamous	YD10B	0.508	0.304	Thyroid Cancer	Vandetanib	MUT + EXP 0.506
Leukemia	ALL	KE37	0.618	0.299	Kidney Cancer	Temsirolimus	MUT + HIST 0.623
		MOLT16	0.603				MUT + EXP 0.605

<table>
<tr><th colspan="4">Empirical</th><th colspan="3">Prescribed</th><th rowspan="2">Top Model: Data Type(s) & Prediction</th></tr>
<tr><th>Primary Disease</th><th>Lineage Subtype</th><th>Cell Line</th><th>AAC</th><th>Highest AAC</th><th>Cancer</th><th>Drug</th></tr>
<tr><td rowspan="9"></td><td></td><td>SEM</td><td>0.657</td><td>0.282</td><td>Pancreatic Cancer</td><td>Sunitinib</td><td>PROT + MIRNA 0.661</td></tr>
<tr><td rowspan="8">AML</td><td rowspan="8">EOL1</td><td>0.992</td><td>0.379</td><td>Colon/Colorectal Cancer</td><td>Regorafenib</td><td>MUT + PROT 0.991</td></tr>
<tr><td>0.991</td><td>0.495</td><td>Thyroid Cancer</td><td>Cabozantinib</td><td>PROT + MIRNA 0.928</td></tr>
<tr><td>0.988</td><td>0.385</td><td rowspan="2">Kidney Cancer</td><td>Sorafenib</td><td>PROT + MIRNA 0.99</td></tr>
<tr><td>0.981</td><td>0.554</td><td>Axitinib</td><td>PROT + RPPA 0.913</td></tr>
<tr><td>0.967</td><td>0.253</td><td>Sarcoma</td><td>Pazopanib</td><td>MUT + PROT 0.816</td></tr>
<tr><td>0.952</td><td>0.282</td><td>Pancreatic Cancer</td><td>Sunitinib</td><td>MUT + PROT 0.941</td></tr>
<tr><td>0.583</td><td>0.304</td><td>Thyroid Cancer</td><td>Vandetanib</td><td>MUT + PROT 0.569</td></tr>
</table>

Empirical				Prescribed			Top Model: Data Type(s) & Prediction
Primary Disease	Lineage Subtype	Cell Line	AAC	Highest AAC	Cancer	Drug	
		KASUMI1	0.659	0.282	Pancreatic Cancer	Sunitinib	CNV + EXP 0.695
			0.647	0.385	Kidney Cancer	Sorafenib	PROT + RPPA 0.674
			0.576	0.253	Sarcoma	Pazopanib	PROT + HIST 0.547
		MOLM13	0.855	0.495	Thyroid Cancer	Cabozantinib	MUT + PROT 0.843
			0.839	0.282	Pancreatic Cancer	Sunitinib	MUT + RPPA 0.841
			0.793	0.385	Kidney Cancer	Sorafenib	MUT + RPPA 0.802
			0.504	0.299		Temsirolimus	PROT + MIRNA 0.505
		MOLM16	0.704	0.495	Thyroid Cancer	Cabozantinib	CNV + MIRNA 0.62

<table>
<tr><th colspan="4">Empirical</th><th colspan="3">Prescribed</th><th rowspan="2">Top Model: Data Type(s) & Prediction</th></tr>
<tr><th>Primary Disease</th><th>Lineage Subtype</th><th>Cell Line</th><th>AAC</th><th>Highest AAC</th><th>Cancer</th><th>Drug</th></tr>
<tr><td rowspan="6"></td><td rowspan="5"></td><td>MONOMAC1</td><td>0.541</td><td>0.299</td><td rowspan="2">Kidney Cancer</td><td>Temsirolimus</td><td>EXP + MIRNA 0.542</td></tr>
<tr><td rowspan="3">MV411</td><td>0.886</td><td>0.385</td><td>Sorafenib</td><td>METAB + RPPA 0.838</td></tr>
<tr><td>0.829</td><td>0.282</td><td>Pancreatic Cancer</td><td>Sunitinib</td><td>CNV + EXP 0.842</td></tr>
<tr><td>0.655</td><td rowspan="5">0.299</td><td rowspan="5">Kidney Cancer</td><td rowspan="5">Temsirolimus</td><td>METAB 0.644</td></tr>
<tr><td>THP1</td><td>0.515</td><td>MUT + MIRNA 0.515</td></tr>
<tr><td>CLL</td><td>JVM3</td><td>0.745</td><td>CNV + MIRNA 0.732</td></tr>
<tr><td rowspan="2">Lung Cancer</td><td rowspan="2">NSCLC</td><td>CALU3</td><td>0.512</td><td>MIRNA + HIST 0.51</td></tr>
<tr><td>EBC1</td><td>0.543</td><td>MUT + CNV 0.55</td></tr>
</table>

Empirical				Prescribed			Top Model: Data Type(s) & Prediction
**Primary Disease**	**Lineage Subtype**	**Cell Line**	**AAC**	**Highest AAC**	**Cancer**	**Drug**	
		HCC827	0.520	0.304	Thyroid Cancer	Vandetanib	EXP + RPPA + HIST + PROT 0.52
		NCIH1703	0.596	0.282	Pancreatic Cancer	Sunitinib	CNV + PROT 0.644
			0.476	0.253	Sarcoma	Pazopanib	MUT + PROT 0.47
		PC14	0.597	0.304	Thyroid Cancer	Vandetanib	MUT + EXP 0.602
	SCLC	NCIH211	0.550	0.299	Kidney Cancer	Temsirolimus	MUT + METAB 0.544
	Mesothelioma	MSTO211H	0.621				CNV + MIRNA 0.614
		NCIH2452	0.580				MIRNA + METAB 0.553

Empirical				Prescribed			Top Model: Data Type(s) & Prediction
**Primary Disease**	**Lineage Subtype**	**Cell Line**	**AAC**	**Highest AAC**	**Cancer**	**Drug**	
Lymphoma	Hodgkin Lymphoma	L540	0.545				MUT + MIRNA 0.546
	Non-Hodgkin Lymphoma	HT	0.616				CNV + MIRNA 0.621
		KARPAS299	0.596				MIRNA + METAB 0.594
		KIJK	0.659				MUT + EXP 0.658
		NUDUL1	0.629				MUT + EXP 0.63
		RI1	0.704				CNV + EXP 0.706
			0.699	0.496	Leukemia	Ibrutinib	CNV + EXP 0.595
		SR786	0.612	0.299	Kidney Cancer	Temsirolimus	MUT + CNV 0.614

Empirical				Prescribed			Top Model: Data Type(s) & Prediction
Primary Disease	Lineage Subtype	Cell Line	AAC	Highest AAC	Cancer	Drug	
		SUDHL4	0.540				CNV + PROT 0.544
		SUDHL6	0.746				MIRNA + RPPA 0.746
			0.725	0.477	Leukemia	Idelalisib	CNV + EXP 0.538
		SUDHL8	0.617	0.299	Kidney Cancer	Temsirolimus	EXP + METAB 0.611
		WSUDLCL2	0.676				EXP + MIRNA 0.669
Myeloma	Multiple Myeloma	KE97	0.589				MIRNA + RPPA 0.593
		KMS11	0.515				EXP + RPPA + HIST + PROT 0.518

Empirical				Prescribed			Top Model: Data Type(s) & Prediction
**Primary Disease**	**Lineage Subtype**	**Cell Line**	**AAC**	**Highest AAC**	**Cancer**	**Drug**	
		KMS12BM	0.539				EXP + PROT 0.537
		KMS21BM	0.669				CNV 0.686
		KMS26	0.837				MIRNA + METAB 0.778
		KMS34	0.824				MUT + MIRNA 0.818
		L363	0.650				MIRNA + HIST 0.645
		MM1S	0.523				MUT + EXP 0.522
Ovarian Cancer	Ovary Adenocarcinoma	A2780	0.622				CNV + EXP 0.621
		OVK18	0.537				CNV + RPPA 0.537

Empirical				Prescribed			Top Model: Data Type(s) & Prediction
**Primary Disease**	**Lineage Subtype**	**Cell Line**	**AAC**	**Highest AAC**	**Cancer**	**Drug**	
Pancreatic Cancer	Exocrine	PATU8988S	0.510	0.304	Thyroid Cancer	Vandetanib	MIRNA + METAB 0.313
Rhabdoid	Malignant Rhabdoid Tumor	A204	0.597	0.282	Pancreatic Cancer	Sunitinib	CNV + PROT 0.609
			0.473	0.253	Sarcoma	Pazopanib	CNV + EXP 0.424
Skin Cancer	Melanoma	COLO829	0.520	0.293	Lung Cancer	Dabrafenib	PROT + RPPA 0.533
		HT144	0.521				CNV + EXP 0.493
		SH4	0.674				MUT + EXP 0.604
		SKMEL24	0.622				CNV + EXP 0.565
		UACC257	0.613				CNV + EXP 0.624

Empirical				Prescribed			Top Model: Data Type(s) & Prediction
Primary Disease	Lineage Subtype	Cell Line	AAC	Highest AAC	Cancer	Drug	
		WM115	0.755				EXP + PROT 0.793
		WM2664	0.575				MUT + MIRNA 0.572
		WM983B	0.578				CNV + EXP 0.572
Thyroid Cancer	Thyroid Squamous	SW579	0.531	0.282	Pancreatic Cancer	Sunitinib	CNV + EXP 0.54
			0.469	0.253	Sarcoma	Pazopanib	MUT + EXP 0.331

Supplementary Table 5. Atomic (node) features for the AttentiveFP model.

Atom feature	Size	Description
Atom symbol	13	['As', 'B', 'Br', 'C', 'Cl', 'F', 'We', 'N', 'O', 'P', 'Pt', 'S', 'other'] (one-hot)
Degree	6	Number of covalent bonds [0, 1, 2, 3, 4, 5] (one-hot)
Formal charge	1	Electrical charge (integer)
Radical electrons	1	Number of radical electrons (integer)

Hybridization	7	[sp, sp2, sp3, sp3d, sp3d2, unspecified, other] (one-hot)
Aromaticity	1	whether the atom is part of an aromatic system [0/1] (one-hot)
Hydrogens	5	number of connected hydrogens [0,1,2,3,4] (one-hot)
Chirality	1	whether the atom is chiral center [0/1] (one-hot)
Chirality type	2	[R, S] (one-hot)

Supplementary Table 6. Bond features for the AttentiveFP model.

Bond feature	Size	Description
Bond type	4	[single, double, triple, aromatic] (one-hot)
Conjugation	1	whether the bond is conjugated [0/1] (one-hot)
Ring	1	whether the bond is in a ring [0/1] (one-hot)
Stereo	4	[StereoNone, StereoAny, StereoZ, StereoE] (one-hot)

Supplementary Table 7. Proportion of bimodal validation samples with predictions within 0.2 of the ground truth AAC target.

		Target Below 0.7		Target Above 0.7	
Data Type(s)	Configuration	Untargeted Drug	Targeted Drug	Untargeted Drug	Targeted Drug
MUT	Baseline	0.9296	0.9156	0.5691	0.0000
	Trifecta	0.9270	0.9158	0.6169	0.0222
CNV	Baseline	0.9692	0.9657	0.6912	0.0000

Data Type(s)	Configuration	Target Below 0.7		Target Above 0.7	
		Untargeted Drug	Targeted Drug	Untargeted Drug	Targeted Drug
	Trifecta	0.9695	0.9639	0.7625	0.1111
EXP	Baseline	0.9723	0.9678	0.7566	0.0444
	Trifecta	0.9766	0.9731	0.7816	0.1556
PROT	Baseline	0.9688	0.9612	0.7287	0.0000
	Trifecta	0.9711	0.9672	0.7353	0.0889
MIRNA	Baseline	0.9684	0.9602	0.6949	0.0000
	Trifecta	0.9661	0.9581	0.7228	0.0222
METAB	Baseline	0.9667	0.9611	0.7015	0.0000
	Trifecta	0.9625	0.9525	0.7000	0.0222
HIST	Baseline	0.9638	0.9541	0.6125	0.0000
	Trifecta	0.9628	0.9547	0.6478	0.0000
RPPA	Baseline	0.9718	0.9634	0.7265	0.0000
	Trifecta	0.9640	0.9582	0.7169	0.0000

Supplementary Table 8. Proportion of trimodal validation samples with predictions within 0.2 of the ground truth AAC target.

Data Type(s)	Configuration	Target Below 0.7		Target Above 0.7	
		Untargeted Drug	Targeted Drug	Untargeted Drug	Targeted Drug
CNV + EXP	Baseline	0.9703	0.9658	0.7434	0.0222
	Trifecta	0.9930	0.9924	0.9375	0.7778
CNV + HIST	Baseline	0.9704	0.9669	0.7316	0.0222
	Trifecta	0.9794	0.9734	0.8346	0.2444
CNV + METAB	Baseline	0.9698	0.9622	0.7419	0.0889
	Trifecta	0.9825	0.9797	0.8625	0.3333
CNV + MIRNA	Baseline	0.9712	0.9656	0.7176	0.0667
	Trifecta	0.9852	0.9833	0.8669	0.4667
CNV + PROT	Baseline	0.9675	0.9601	0.6515	0.0000
	Trifecta	0.9915	0.9909	0.9331	0.6222
CNV + RPPA	Baseline	0.9698	0.9657	0.6978	0.0000
	Trifecta	0.9834	0.9818	0.8779	0.5333
EXP + HIST	Baseline	0.9729	0.9697	0.7787	0.0889
	Trifecta	0.9807	0.9794	0.8331	0.2000

Data Type(s)	Configuration	Target Below 0.7		Target Above 0.7	
		Untargeted Drug	Targeted Drug	Untargeted Drug	Targeted Drug
EXP + METAB	Baseline	0.9722	0.9672	0.7647	0.0889
	Trifecta	0.9851	0.9837	0.8824	0.4222
EXP + MIRNA	Baseline	0.9716	0.9668	0.7684	0.0889
	Trifecta	0.9813	0.9776	0.8228	0.1556
EXP + PROT	Baseline	0.9644	0.9513	0.6912	0.0222
	Trifecta	0.9895	0.9873	0.9051	0.5778
EXP + RPPA	Baseline	0.9713	0.9662	0.7684	0.1556
	Trifecta	0.9870	0.9883	0.8831	0.4444
HIST + RPPA	Baseline	0.9676	0.9657	0.7074	0.0444
	Trifecta	0.9829	0.9766	0.8663	0.3095
METAB + HIST	Baseline	0.9691	0.9610	0.6949	0.0000
	Trifecta	0.9698	0.9618	0.7500	0.0444
METAB + RPPA	Baseline	0.9713	0.9661	0.7368	0.0222
	Trifecta	0.9721	0.9705	0.7963	0.3111
MIRNA + HIST	Baseline	0.9685	0.9642	0.6831	0.0000
	Trifecta	0.9826	0.9756	0.8667	0.2500

Data Type(s)	Configuration	Target Below 0.7		Target Above 0.7	
		Untargeted Drug	Targeted Drug	Untargeted Drug	Targeted Drug
MIRNA + METAB	Baseline	0.9699	0.9642	0.7037	0.0000
	Trifecta	0.9889	0.9855	0.9272	0.6000
MIRNA + RPPA	Baseline	0.9696	0.9642	0.7140	0.0222
	Trifecta	0.9868	0.9843	0.8918	0.5946
MUT + CNV	Baseline	0.8891	0.8778	0.6441	0.0222
	Trifecta	0.9878	0.9847	0.8934	0.5556
MUT + EXP	Baseline	0.9233	0.9163	0.6463	0.0222
	Trifecta	0.9923	0.9919	0.9257	0.6889
MUT + HIST	Baseline	0.9417	0.9263	0.6118	0.0000
	Trifecta	0.9808	0.9777	0.8250	0.2667
MUT + METAB	Baseline	0.9403	0.9274	0.6603	0.0000
	Trifecta	0.9852	0.9790	0.8581	0.3778
MUT + MIRNA	Baseline	0.9272	0.9135	0.6110	0.0000
	Trifecta	0.9861	0.9826	0.8882	0.4889
MUT + PROT	Baseline	0.9239	0.9066	0.5809	0.0000
	Trifecta	0.9908	0.9894	0.9007	0.6444

Data Type(s)	Configuration	Target Below 0.7		Target Above 0.7	
		**Untargeted Drug**	**Targeted Drug**	**Untargeted Drug**	**Targeted Drug**
MUT + RPPA	Baseline	0.9269	0.9126	0.5941	0.0000
	Trifecta	0.9849	0.9846	0.8691	0.5778
PROT + HIST	Baseline	0.9687	0.9580	0.7081	0.0000
	Trifecta	0.9869	0.9836	0.8963	0.4000
PROT + METAB	Baseline	0.9671	0.9575	0.6721	0.0000
	Trifecta	0.9901	0.9877	0.9235	0.5556
PROT + MIRNA	Baseline	0.9655	0.9534	0.6551	0.0222
	Trifecta	0.9906	0.9899	0.9353	0.7111
PROT + RPPA	Baseline	0.9683	0.9603	0.7221	0.0000
	Trifecta	0.9900	0.9881	0.9324	0.7778

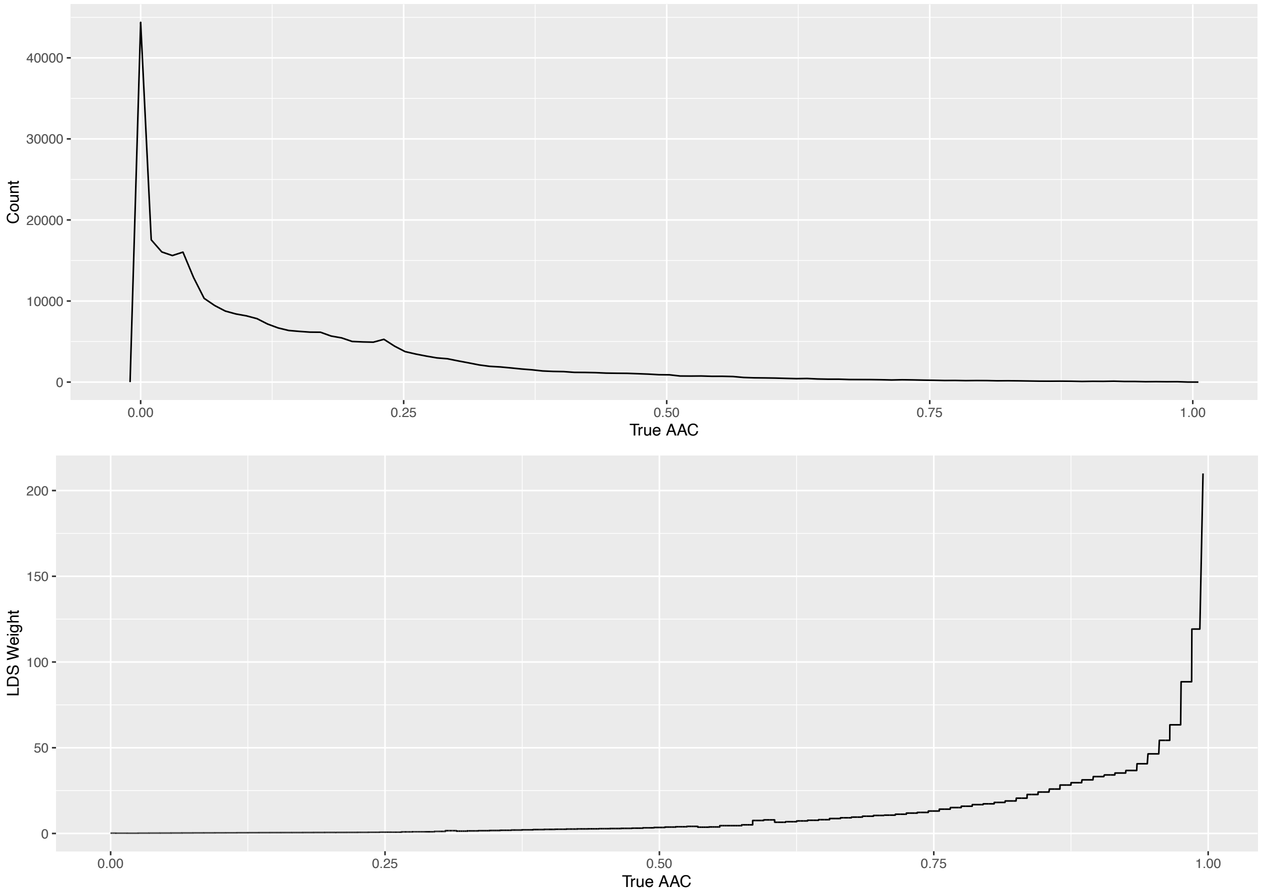

Supplementary Figure 1. Distribution of AAC (top) vs LDS-calculated weights (bottom) in the CTRPv2 dataset. LDS assigns higher weights to less frequently observed samples, which in the case of CTRPv2 also have higher AACs. The LDS weight distribution is roughly the inverse of the AAC distribution.

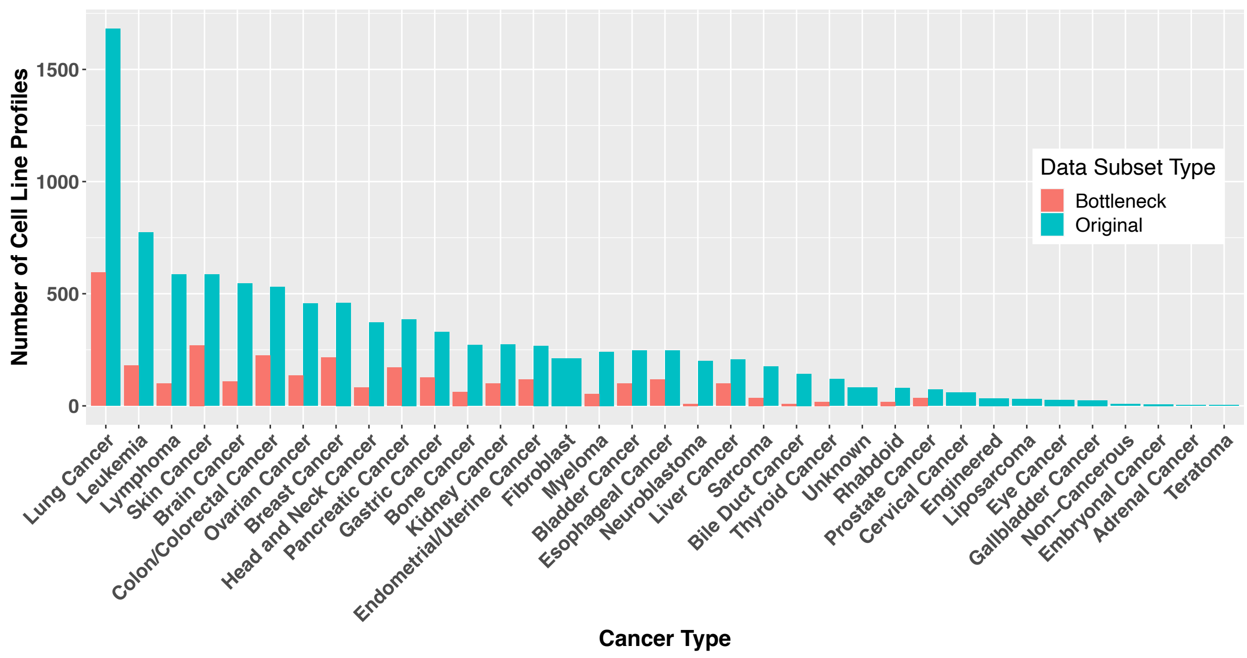

Supplementary Figure 2. The subset of complete samples by cancer types in DepMap. Some cancer types do not have cell lines that are characterized with all eight omic profiling technologies. Because of this, they are not represented in the bottlenecked dataset.

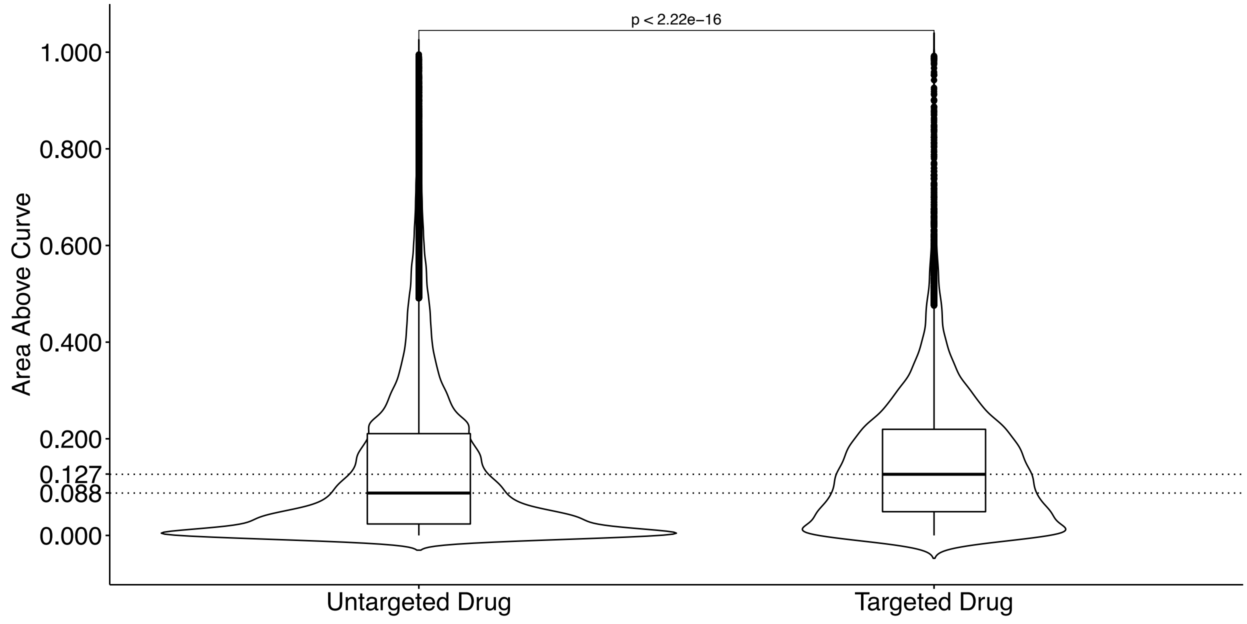

Supplementary Figure 3. Violin plot and boxplot comparison of AAC distributions of samples with targeted (n=20,842) and untargeted (n=289,950) drugs. The violin plot depicts the density at each AAC, whereas the

boxplot indicates the lower quartile, median and upper quartile of the AAC distributions for targeted and untargeted drugs. Dotted lines indicate the medians of each distribution. The median AAC for targeted drugs is 0.127, which is higher than the median AAC for untargeted drugs at 0.088 (p-value < 2.22×10^{-16}, Mann-Whitney U Test).

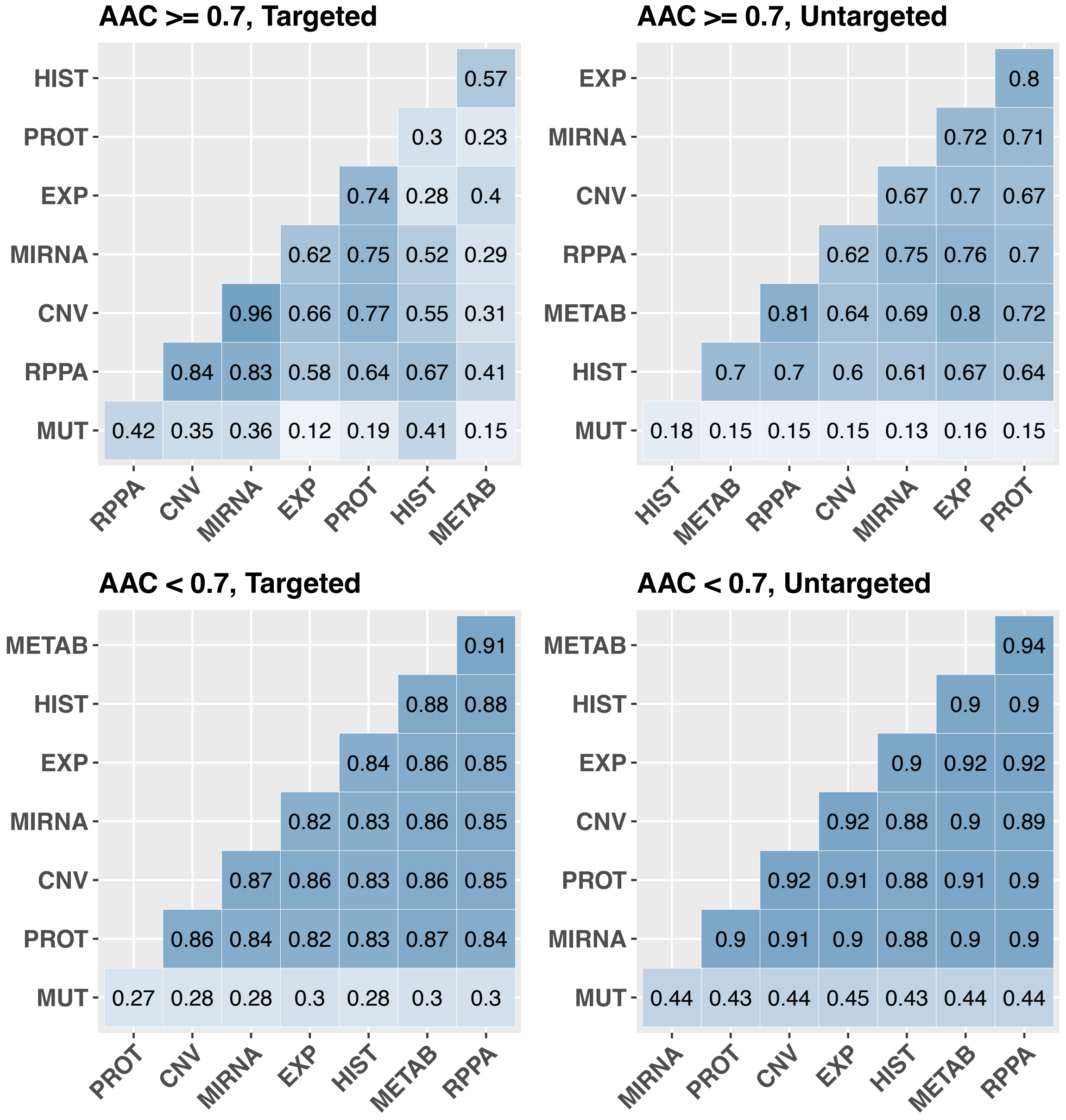

Supplementary Figure 4. R2 measure of agreement between predictions of bimodal baseline models in distinct AAC range and drug type subsets. The agreement between different models in the higher AAC ranges, especially in samples with targeted drugs, is much less than in other range and drug type. This indicates the certain

omic profiling data may be more useful for specific cell line and drug combinations, and for the mechanisms of action of targeted drugs.

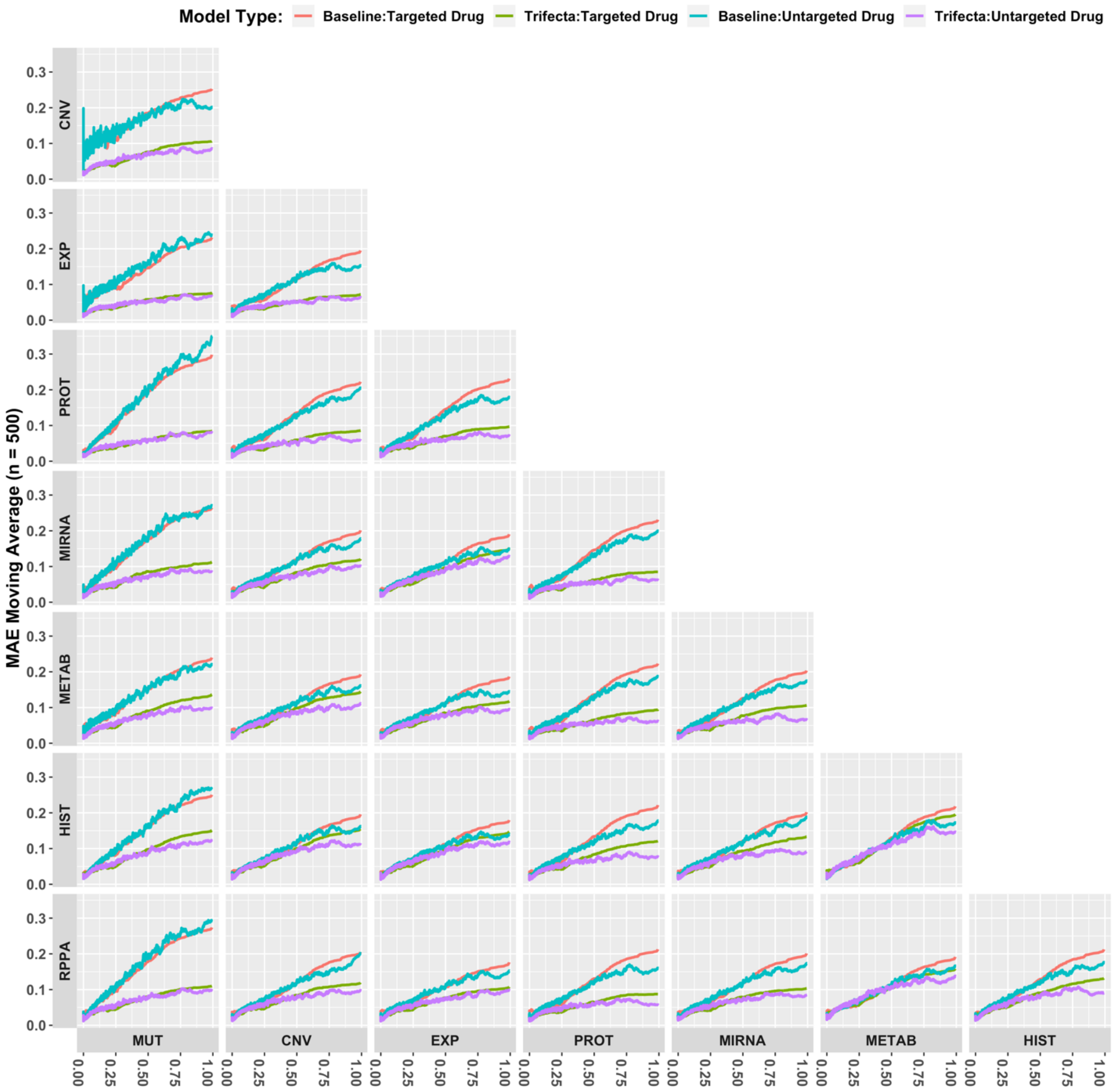

Supplementary Figure 5. Comparison of trimodal baseline vs. trifecta models. Trailing moving average of MAE losses in baseline and trifecta models by drug type in each sub-plot. The data types on the horizontal and vertical axes comprise the two omic data types used in each trimodal model. The trifecta configuration allows for a better performance in both targeted and untargeted drugs, across almost all trimodal data combinations. Untargeted drugs remain easier to predict than targeted drugs across all configurations and data types.

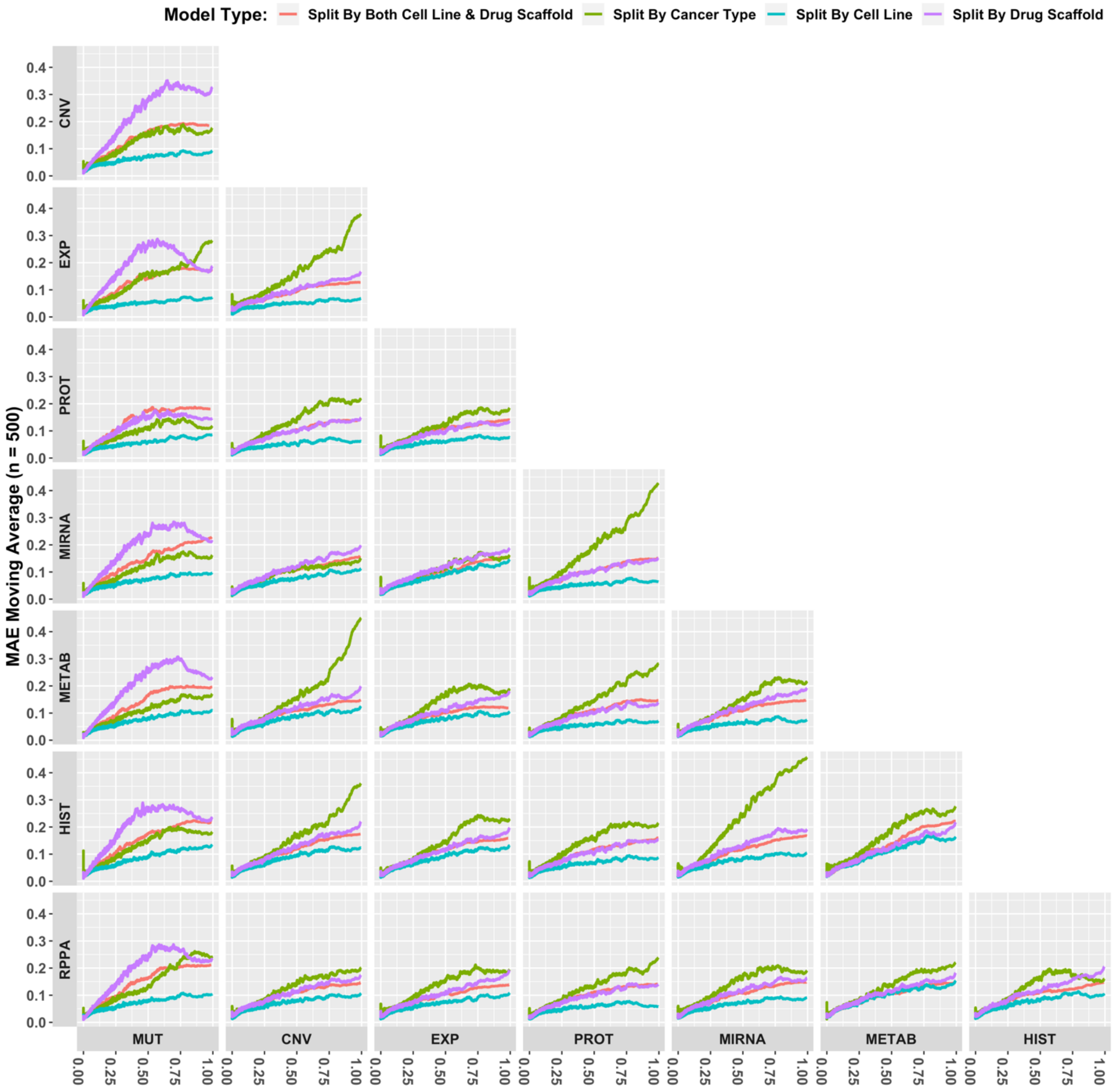

Supplementary Figure 6. Comparison of different splitting methods in trimodal trifecta models. Often, the Split by Cancer Type has the highest losses, indicating that the generalization to novel cancer types is the most difficult task among those tested.

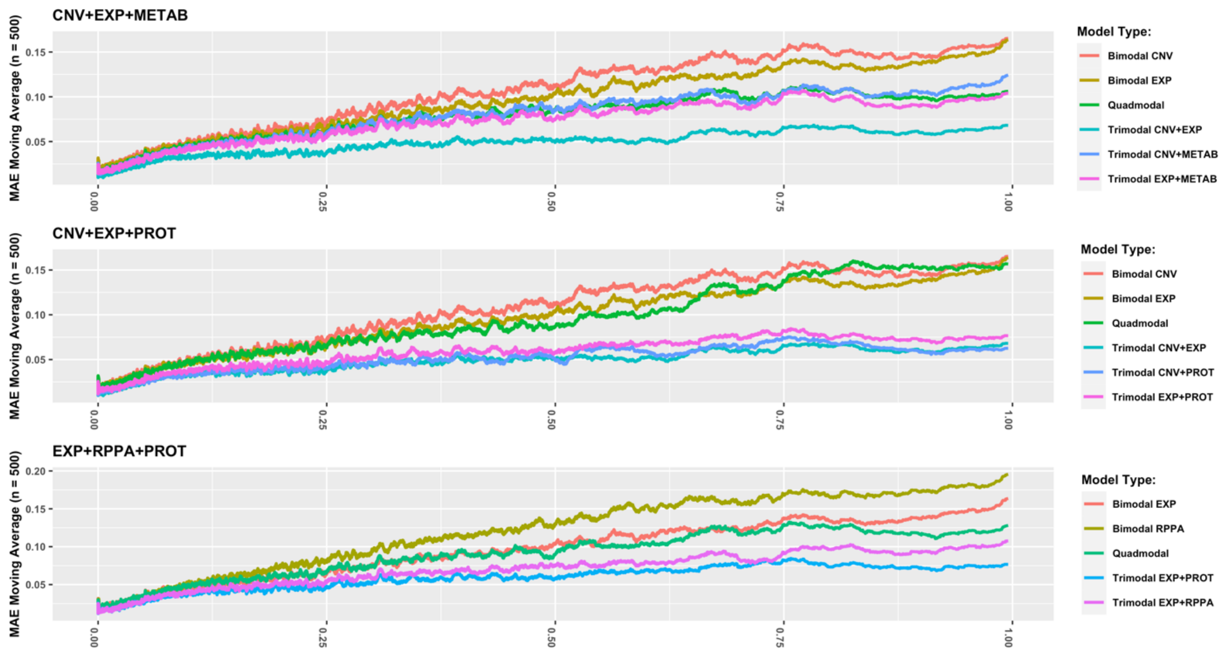

Supplementary Figure 7. Comparison of quadmodal models with their trimodal and bimodal counterparts. Trimodal models, which use two omic datatypes that comprise a subset of the three omic datatypes used by the quadmodal models, have a better performance.

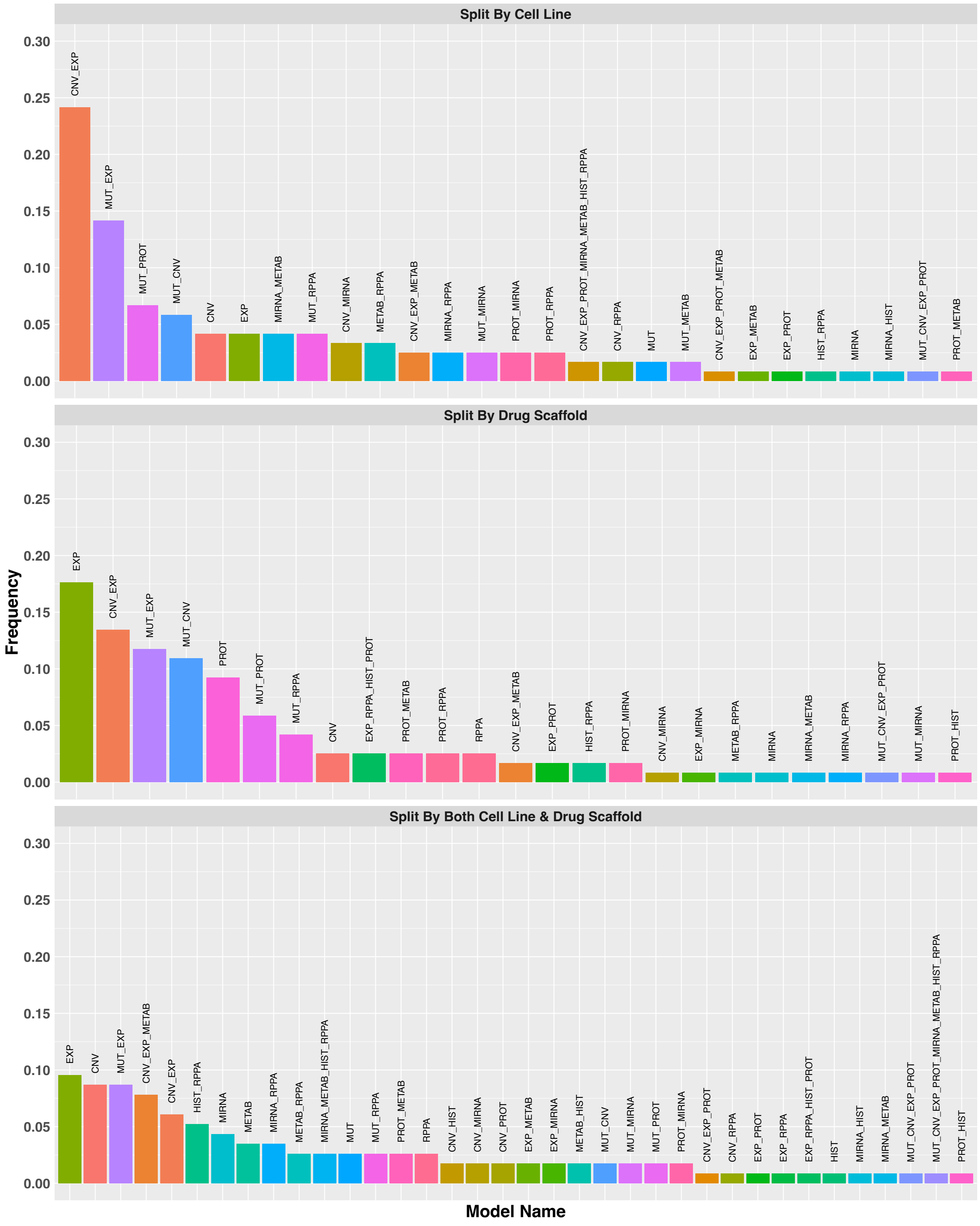

Supplementary Figure 8. Frequency of omic data types used by models that predict AAC most accurately for each cell line and drug combination, divided by splitting schemes.

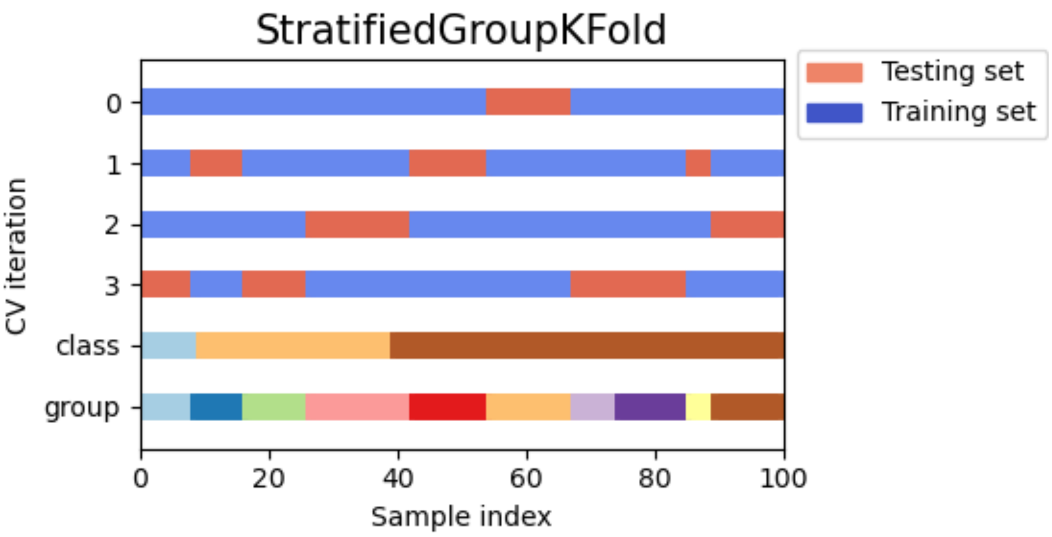

Supplementary Figure 9. Stratified Group k-fold cross-validation data splitting scheme. This scheme considers class and group distributions, and divides training and validation folds such that they more closely represent the original class and group distributions. This helps prevent biases in performance assessment. This figure was taken from: Visualizing cross-validation behavior in scikit-learn. https://scikit-learn.org/stable/auto_examples/model_selection/plot_cv_indices.html#sphx-glr-auto-examples-model-selection-plot-cv-indices-py[60]
